# Comparative study on immunoglobulin Y transfer from breeding hens to egg yolk and progeny chicks in different breeds of poultry

**DOI:** 10.14202/vetworld.2016.425-431

**Published:** 2016-04-30

**Authors:** Ritu Agrawal, S. D. Hirpurkar, C. Sannat, Amit Kumar Gupta

**Affiliations:** Department of Veterinary Microbiology, College of Veterinary Science & Animal Husbandry, Anjora, Durg, Chhattisgarh, India

**Keywords:** breeding hens, chicks, maternal immunoglobulin Y, *Salmonella* antibody, yolk

## Abstract

**Aim:**

This study was undertaken to compare the immunoglobulin Y (IgY) level and its efficacy in laying hens of four different breeds of poultry (viz., Vanraja, Gramapriya, BlackRock, and KalingaBrown) and its relative transfer in egg yolk and chick.

**Materials and Methods:**

This study was conducted in 48 apparently healthy laying hens vaccinated with Salmonella inactivated polyvalent vaccine, eggs and progeny chicks; 12 each from four different breeds of poultry, viz., Vanraja, Gramapriya, BlackRock, and KalingaBrown. The methodology included measurement of egg and yolk weight, total protein and IgY in egg yolk, total serum protein and IgY in breeding hens, and progeny chicks and extent of IgY transfer from hens to yolk then to chicks. Further, Salmonella-specific antibodies in breeding hens, egg yolk and progeny chicks were assessed using O and H antigen by tube agglutination test.

**Results:**

The egg weight differed nonsignificantly (p>0.05) among breeds, however, breed wise significant variation (p<0.01) was reported in yolk weight. The weight of egg yolk significantly affects the total protein and IgY concentration although these levels per unit of volume did not differ. Total protein was significantly higher (p<0.01) in KalingaBrown and Gramapriya as compared to Vanraja and BlackRock. Non-significant (p>0.05) difference among breed was found in total protein of egg yolk and chick. The IgY concentration in hens, egg yolk and chick was found to be in the range of 5.35±0.63-5.83±0.65, 2.3±0.1-2.6±0.2, and 1.3±0.11-1.7±0.16 mg/ml, respectively which is uniform and independent of total protein concentration at all the three levels. Significant breed variations were not observed in maternal IgY transfer from breeding hens to chicks and were 25.62±1.42-36.06±4.34% of total IgY in parent flock. Moderate to higher rate of seroprevalence with peak titers of 1:640 against Salmonella-specific antibodies was observed in only 41.6% of breeding hens.

**Conclusion:**

No significant difference in the rate of transfer of IgY was observed in four breeds studied (viz., Vanraja, Gramapriya, BlackRock, and KalingaBrown) and moderate seropositivity was detected for Salmonella-specific antibodies in progeny chicks.

## Introduction

Chicken of all age groups are susceptible to many pathogens if innate immune response by maternal antibody transfer and/or active immune response by foreign materials (vaccine) are not evoked at its full potential [1]. In general, advanced poultry production practice promises full protection by immunization which is cleverly designed by a combination of breeder hen vaccination and active immunization of chicks at appropriate age during early life. Efficacy of breeder hen vaccination is adjudged by quantum of maternal antibodies received by progeny chicks from dam and thereby newly hatched chicks are protected from diseases even if they lack fully developed immune system. The importance of vertical transmission of immunity to provide specific pathogen protection during the early post-hatching period has long been recognized. Immunoglobulin (Ig)-secreting B cells of chick origin have been detected in circulation after 6 days post-hatch [2], meaning that during the first days of the post-hatching period, humoral immunity is totally dependent on the maternal transfer of Igs. In the domestic chicken, 3 classes of Igs have been identified as the homologs of mammalian IgM, IgA, and IgG. Avian IgY is the evolutionary ancestor of mammalian IgG and is the main defense mechanism against systemic infections [3]. Contrary to mammals, who after birth may obtain maternal antibodies in the colostrums, all of the maternal Igs needed to protect the newly hatched chick must be incorporated into the egg before it is laid.

The transfer of IgY from the hen to the chicks is completed in two steps, first transfer of circulating IgY from the hen’s bloodstream into the ovarian follicle (i.e., the egg yolk) and then to the embryo[4]. The natural transfer of antibodies that occurs from hen to chick via the egg yolk can be exploited to produce antibodies specific to a given pathogen, simply by immunizing the laying hens with an antigen from this targeted pathogen [5]. However, despite seropositivity and the presence of maternal antibodies, some of the poultry farms face the problem of disease outbreak, particularly when the pathogen has potential of vertical transmission. Salmonellosis is one such problem which intensifies many folds due to emerging antibiotic resistant *Salmonella* spp. and increasing zoonotic threat to human population [6]. Thus, overall transfer of maternal antibody and its efficacy depends mainly on individual titers of specific antibody coupled with rate at which prevalence of the pathogen is recorded.

The present study was therefore planned with a view compare the IgY level in laying hens of four different breeds of poultry (*viz*. Vanraja, Gramapriya, BlackRock, and KalingaBrown) and its relative transfer in egg yolk and chick. Finally, the efficacy of IgY transferred was judged by evaluating the presence of specific (*Salmonella*) antibodies.

## Materials and Methods

### Ethical approval

This study was approved by Institutional Animal Ethics Committee of Veterinary College, Anjora, Durg.

### Birds under study

Apparently healthy laying hens (40-60 weeks of age) from four different breeds of poultry, *viz*., Vanraja, Gramapriya, BlackRock, and KalingaBrown were selected on a random basis and reared under optimum feeding (as per BIS) and management condition at Government Poultry Farm, Durg. No additional supplements like IgY and other growth promoters were added in the feed. These hens were vaccinated with *Salmonella* polyvalent vaccine inactivated (Venky’s). Sufficient numbers of egg were collected, and hatching of eggs was carefully undertaken. Apparently healthy day old chicks from all the four different breeds of poultry were separated immediately after hatching and then used for study within 2-3 h. 12 numbers of samples were studied for each breed of poultry.

### Measurement of weight of egg and egg yolk

About 48 eggs were collected from four different breeds (12 numbers from each breed) of poultry. First, each egg was weighed and then broken so that the yolk could be separated from the egg albumin. The yolk was then separated by rolling over filter paper to remove the albumin adhered to it. Weight of each yolk so collected was recorded in gram.

### Determination of total protein and IgY in egg yolk

Total protein in egg yolk was measured by colorimetric method using Biuret reagent [7] and the results were expressed as mg/ml. The total IgY concentration in egg yolk was assessed after 7 days of serum collection from breeding hens. Polyethylene glycol (PEG)-precipitationtechnique was performed for purification of IgY as per procedure described by Polson *et al*. [8] with slight modifications. The concentration of IgY extract was measured by single radial immunodiffussion (SRID) [9]. Two-fold serial dilution (1:2 to 1:32 dilution equivalent to 11.97, 5.9, 3, 1.5, and 0.74 mg/ml of standard antigen concentration, respectively) of purified chicken IgY at 23.8 mg/ml (BangaloreGenei) was used as standards to generate standard curves by plotting the zone annulus area (the area of zone minus the area of well) of the precipitation rings after 18 h diffusionin a moist chamber at room temperature.

### Determination of total protein and IgY concentration in breeding hens and their progeny chicks

Serum samples were collected from breeding hens and chicks under study and stored at −20°C in deep freeze. The total IgY concentration in chicks was recorded on the day of hatch. The concentration of total serum protein was measured by colorimetric method using Biuret reagent [7], and the results were expressed as g/dl. IgY concentration in serum was estimated using SRID.

### Extent of IgY transfer in four different breeds

Percent transfer of maternal antibody was calculated as below:


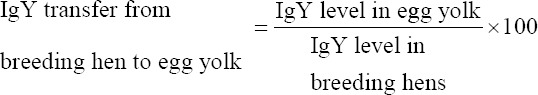



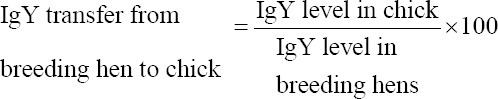


### Assessment of Salmonella-specific antibodies in breeding hens, egg yolk, and progeny chicks

The *Salmonella* antigens, *viz*., Somatic (O) and Flagellar (H) from stock culture were prepared using standard protocol as mentioned in OIE terrestrial manual [10] with slight modifications. Standard titration was carried out with known serum to ensure presence of antigen and stored in a refrigerator at 4°C until required. The presence of *Salmonella*-specific antibodies was detected in serum samples from breeding hens and chicks and PEG extract for egg yolk using tube agglutination test. The highest dilution of the serum sample that gives a visible agglutination was recorded as the titer of the serum using antigen suspensions.

## Results

### Weight of egg and yolk

Although, nonsignificant (p>0.05) variation was reported in the egg weight among four breeds (Table-1), but comparatively higher egg weight was observed in KalingaBrown (56.67±1.42 g) as compared to Vanraja (55±1.51 g). However, the weight of the yolk differs significantly (p<0.01) among the breeds studied. Significantly higher yolk weight was found in BlackRock (18.76±0.40g) as compared to Gramapriya (15.82±0.27 g) and KalingaBrown (17.06±0.38 g).

**Table-1:** Egg weight and yolk weight of four different breeds of poultry.

Particular	Breeds				Level of significance

	Vanraja	Gramapriya	BlackRock	KalingaBrown	
Egg weight (g±SEM)	55±1.51	55.33±1.66	55.83±1.21	56.67±1.42	NS
Yolk weight (g±SEM)	18.04±0.37^ab^	15.82±0.27^c^	18.76±0.40^a^	17.06±0.38^b^	**

abc Means having different superscripts in a row differs significantly.

** p<0.01, NS=Not significant, SEM=Standard error of mean

### Total protein

Total protein (Table-2) in breeding hens was found to be significantly higher (p<0.01) in KalingaBrown and Gramapriya (5.69±0.41 and 5.18±0.31 g/dl, respectively) as compared to Vanraja and BlackRock (3.54±0.30 and 3.76±0.30, respectively). Nonsignificant (p>0.05) difference in total protein was recorded in egg yolk (164±1.82-175±6.03 mg/ml) and chick (2.03±0.10-2.38±0.16 g/dl).

**Table-2:** Total protein levels in breeding hens, egg yolk and chicks in four different breeds of poultry.

Source of samples	Breeds				Level of significance

	Vanraja	Gramapriya	BlackRock	KalingaBrown	
Breeding hens (g/dl±SEM)	3.54±0.30^b^	5.18±0.31^a^	3.76±0.30^b^	5.69±0.41^a^	**
Egg yolk (mg/ml±SEM)	168±7.94	164±1.82	175±6.03	171.62±5.81	NS
Chick (g/dl±SEM)	2.27±0.11	2.30±0.07	2.38±0.16	2.03±0.10	NS

ab Means having different superscripts in a row differs significantly. NS=Not significant;

** Highly significant (p<0.01), SEM=Standard error of mean

### IgY concentration

Nonsignificant (p>0.05) differences in IgY concentration were recorded among different breeds of poultry. The IgY concentration ranged from 5.35±0.63-5.83±0.65, 2.3±0.1-2.6±0.2, and 1.3±0.11-1.7±0.16 mg/ml in hens, egg yolk, and chicks, respectively (Table-3).

**Table-3:** IgY levels in breeding hens, egg yolk and chicks in four different breeds of poultry.

Source of samples	Breeds				Level of significance

	Vanraja	Gramapriya	BlackRock	KalingaBrown	
Breeding hens (mg/ml±SEM)	5.83±0.65	5.35±0.63	5.42±0.70	5.59±0.69	NS
Egg yolk (mg/ml±SEM)	2.4±0.1	2.3±0.1	2.6±0.2	2.5±0.3	NS
Chick (mg/ml±SEM)	1.5±0.16	1.3±0.11	1.7±0.16	1.5±0.08	NS

NS=Not significant, SEM=Standard error of mean, IgY=Immunoglobulin Y

### Extent of IgY transfer in four different breeds

The extent of IgY transfer were nonsignificant (p>0.05) among different breeds (Table-4). The transfer of IgY from breeding hens to egg yolk, egg yolk to chick and breeding hens to chick were in range of 46.39±1.42-54.92±5.3%, 54.39±1.93-66.52±1.99%, and 25.62±1.42-36.06±4.34%, respectively.

**Table-4:** Percentage transfer of IgY from breeding hens to egg yolk and chicks in four different breeds of poultry.

Sources of samples	Breed wise transfer (%±SEM)				Level of significance

	Vanraja	Gramapriya	BlackRock	KalingaBrown	
Breeding hens to egg yolk	46.39±1.42	51.10±3.39	54.92±5.3	54.86±6.1	NS
Egg yolk to chicks	60.46±3.70	54.39±1.93	66.52±1.99	58.09±2.21	NS
Breeding hens to chicks	26.84±1.82	25.62±1.42	36.06±4.34	30.34±2.45	NS

NS=Not significant, SEM=Standard error of mean, IgY=Immunoglobulin Y

### Salmonella-specific passive transfer of IgY

In this study, 79.16% of the serum samples from breeding hens were found to be seropositive for O antigen while 18.75% for H antigen (Table-5). In fact, only 2-10% of total specific antibody in hens is transferred to chicks. Titer of Specific antibodies against *Salmonella* in breeding hens among different breeds is shown in Table-6. More than 1:640 antibody titer for O antigen was recorded in 41.6% (20) samples, however, only 8% antibody titer was observed for H antigen.

**Table-5:** Seroprevalence of specific antibodies against *Salmonella* in breeding hens.

Breed	Total number of samples	Number of samples positive		Percentage positive	
	
		O antigen	H antigen	O antigen	H antigen
Vanraja	12	10	4	83.3	33.3
Gramapriya	12	11	3	91.7	25.0
BlackRock	12	9	2	75	16.6
KalingaBrown	12	8	UD	66.6	-
Total	48	38	9	79.16	18.75

UD=Undetectable

**Table-6:** Titer of specific antibodies against *Salmonella* in breeding hens in different breeds.

Antibody titre	Number of samples positive (%)							

	Vanraja		Gramapriya		BlackRock		KalingaBrown	
			
	O antigen	H antigen	O antigen	H antigen	O antigen	H antigen	O antigen	H antigen
640	5 (41.6)	-	7 (58.3)	-	7 (58.3)	1 (8)	1 (8)	-
320	3 (25)	4 (33.3)	2 (16.6)	3 (25)	2 (16.6)	1 (8)	4 (33.3)	-
160	2 (16.6)	-	2 (16.6)	3 (25)	-	3 (25)	3 (25)	-
80	1 (8)	3 (25)	1 (8)	3 (25)	2 (16.6)	2 (16.6)	-	2 (16.6)
<80	1 (8)	5 (41.6)	-	3 (25)	1 (8)	5 (41.6)	4 (33.3)	10 (83.33)

Total number of samples from each breeds 12

### Data recording and statistical analysis

Data were analyzed by applying general linear model for factorial experiments using SPSS computer software package (Version 16.0.0.247^©^ 2007). Duncan’s multiple range tests were done to make specific treatment comparisons for values that were found significant by ANOVA.

## Discussion

The difference in the total protein concentration in different breeds of breeding hen may be due to difference in the genetic makeup as the total serum protein is influenced by breed, age, physiological state, environment and antigen exposure and the levels can be extremely variable [11]. Values of serum protein concentration in breeding hens are well supported by El-Sheikh *et al*. [12]. On contrary, slightly lower values were reported by Malakian *et al*. [13]. The findings of total serum protein in chicks (2.03±0.10-2.38±0.16) are also in accordance with those reported by Bowes *et al*. [14]. Low serum protein in chicks as compared to hens in this study are in accordance with findings of Kaneko [15], who observed that concentration of protein is significantly lower in young animals than in adults. Some relevant reports revealed that the total proteins are the yolk precursors, which are synthesized in the liver and transported via the plasma to the ovary where they were incorporated in the oocyte [16]. In contrast to present observation, Li *et al*. [17] reported higher values of protein in egg yolk.

The IgY concentration in hens and chicks during present work are in congruence with the findings of Hamal *et al*.[18]. In agreement with present observation, Carlander *et al*. [19] also reported different levels of IgY/mL of egg yolk among strains, however, higher values (9.3-11.3 mg/g) were recorded by Ulmer-Franco *et al.*, [20]. Immunization of hens yielded higher IgY level in yolk [21]. Likewise present finding, Sun *et al.*, [22] reported significant correlation among IgY levels in hen serum, yolk, and offspring serum in White Leghorn, Silkie, and Dongxiang blue-shelled chickens. Varied concentration of IgY might be attributed to different techniques used for extraction, purification, and concentration of IgY by different authors, as purity of the extract by PEG precipitation technique may be around 80% only [23]. However, age and feeding condition might had no or very little effect on IgY concentration as birds were of uniform age groups and optimum and uniform feeding conditions were applied during present investigation.

As regards the total protein and IgY concentration per ml egg yolk, there was no significant difference. As the total weight of yolk is less as observed in Gramapriya (15.82±0.27 g), there was proportionate decrease in the concentration of total protein and total IgY. Hence, the total protein and IgY concentration per egg yolk were found to be significantly different in four breeds studied.

Yolk weight observed are in accordance with the findings of Niranjan *et al*. [24] who also reported a significant difference in yolk weight of Vanraja and Gramapriya breeds. This is also in conformity with reports of Haunshi *et al*. [25]. Similar to present finding, Li *et al*. [17] observed that weight of yolk have direct effect on total protein and IgY levels. The high levels of IgY might be because of the prior immunization of hens with bovine serum albumin. Differences reported in this study might be due to the difference in the method used, as the PEG extraction method yield high levels of IgY from egg yolk [26]. The IgY concentration by PEG extraction method yielded almost twice than that of other methods.

Very young chicks are susceptible to many pathogens during the first few weeks of age because their immune system is not fully developed; hence, maternal antibodies are the primary means of antigen-specific protection. There are many reports in the literature regarding the transfer of pathogen-specific antibodies from hens to their chicks via the egg, and their role in the protection of newly hatched chicks from the pathogens [18,27]. It was also observed that the amount of IgY deposited in the egg and thereby transferred to the offspring were directly related to the circulating levels of IgY in the dam. During present work, the percentage transfer expressed as the percentage of the dam’s plasma IgY levels circulating in the blood of day-old chicks (approximately 25-36%) did not differ significantly in the four breeds of chickens. This observation suggests that the amount of maternal antibody present in chick was ultimately decided by the levels in the dam and not the breed. However, this is not in conformity with findings of some earlier workers [28] who have detailed variation in 4 native and crossbred chicken lines with respect to the amounts of inherited maternally derived antibodies in both yolk and day-old chicks.

The present observations on percentage transfer of IgY from hens serum to chick fall in range between 25% and 36%. The differences were, however, not significant when compared among the four breeds which collaborate with findings of Hamal *et al*. [18]. Antibody levels in the egg yolk are directly proportional to the antibody levels in the dam’s serum [29]. Thus, understanding on the relationship between maternal antibody transfer and endogenous antibody production in layer chicks, one may find its direct application in formulating strategies for protecting chicks, especially during the first few weeks of age when their own immune systems are not yet fully functional. It is reported that only minor quantities of IgA and IgM are transferred to the egg yolk from the plasma cells of the oviduct [30] and 10-15% of immunized hens might be low responders to certain antigens which correspond less IgY in egg yolk [31].

By immunizing the laying hens with an antigen from targeted pathogen induces efficient protection in chicks [5] but it is short-term and is limited to infections present in the hen’s environment at the time of lay [32]. Usually, titer of 100 or more for O antigen is considered significant, and a titer in excess of 200 for H antigen is considered significant for salmonellosis. Seropositivity for *Salmonella* in this study collaborates with the finding of Betancor *et al*. [33] who found 24.4% of the birds sera to be positive for *Salmonella* group O: 9. It may be attributed to change in geographical conditions because a particular serotype was prevalent in a particular area. The specific antibody could not be detected in egg yolk and chicks by tube agglutination test. In fact, only 2-10% of total specific antibody in hens is transferred to chicks. Moderate to low antibody titer in hens render their chicks with undetectable levels of *Salmonella* antibodies by STAT.

The results revealed as much as cent percent immunity at flock level, out of which, only 41.6% show titer of 1:640 which can be correlated with the reports of Kumar *et al*. [34] in which the antibody titer was found to be highest (1:1280) which decreased gradually, but the bird remain seropositive until end of observation, i.e. 6^th^ week. The results are slightly lower than the findings of Ahmed *et al*.[35] who reported seroprevalence of 49.5% in live birds and the rate decreased with advancement of age. It is opined that due to resultant low titer in hen, antibodies in chicks could not reach to level to be detected by STAT. Likewise, Hermans *et al*.[36] observedtiters of 1:16 000 for *Campylobacter jejuni*-specific IgY in egg yolk of hens immunized with a *C. jejuni* whole-cell lysate. This indicates that transfer of IgY to egg yolk is biologically relevant in the overall transfer of Igs into eggs after immunization of hens.

Multiple research studies using hyperimmunization of hens to produce avian [37] and interspecies-specific egg Ig [38] have been carried out. However, faster absorption of yolk sac IgY in chicks from breeding hens, might led the chicks more susceptible to localized pathogen invasion of the yolk sac during the early post-hatching period. In the post-hatching period, protein integrity within the yolk sac is critical for normal absorption of the yolk sac content and for IgY transfer to the circulation of the chick[39]. Hence, factors affecting transfer of IgY to the chick may threaten the chick’s immune status and increase disease susceptibility during the early post-hatching period.

## Conclusion

This study reports comparative IgY transfer from parent flock to yolk and then to chicks in four breeds of poultry, *viz*., Vanraja, Gramapriya, BlackRock, and KalingaBrown. Extent of IgY transfer among four breeds at three different stages wasnonsignificant, as it may depend on individual dam serum IgY concentration. Moderate to high percent seropositivity was detected for *Salmonella*-specific IgY in hens immunized with polyvalent *Salmonella* vaccine. This work has initiated investigations to assess the cutoff IgY concentration received by progeny from hen that impart specific immunity to infection in the early period of life in the immunocompetent chicks.

## Authors’ Contributions

SDH designed the experiment and RA performed the experiment under supervision of SDH. CS and AKG helped in sample collection and analysis. Manuscript preparation was reviewed and edited by all authors. All authors read and approved the final manuscript.
